# Effect of vitrectomy with silicone oil tamponade and internal limiting membrane peeling on eyes with proliferative diabetic retinopathy

**DOI:** 10.1038/s41598-022-12113-8

**Published:** 2022-05-16

**Authors:** Sung Yeon Jun, Daniel Duck-Jin Hwang

**Affiliations:** 1 Department of Ophthalmology, Hangil Eye Hospital, 35 Bupyeong-daero, Bupyeong-gu, Incheon, 21388 South Korea; 2 Department of Ophthalmology, Catholic Kwandong University College of Medicine, Incheon, South Korea

**Keywords:** Eye diseases, Retinal diseases, Diabetes complications

## Abstract

We investigated the combined effect of silicone tamponade and the internal limiting membrane (ILM) peeling and investigated whether timing of peeling of the ILM affects the outcomes of vitrectomy with silicone oil tamponade in eyes with proliferative diabetic retinopathy (PDR). Here, we examined 63 eyes (58 patients) with PDR, which underwent vitrectomy with silicone oil tamponade and stepwise removal of silicone oil. ILM peeling was performed just before oil injection (group 1; 33 eyes, 30 patients) or after oil removal (group 2; 30 eyes, 28 patients). Visual acuity and retinal and choroidal thicknesses were compared between the groups. Thinning of the inner retina, including the ganglion cell-inner plexiform layer and macular retinal nerve fiber layer, was evident at 1 year after surgery in both groups. Thinning of the total retina (*P* = 0.019) and inner retina (*P* = 0.008) was significantly correlated with final visual acuity. There was no considerable between-group difference observed in final visual acuity, intraocular pressure, or retinal or choroidal thickness at 1 year after surgery. The incidence of epiretinal membrane was higher during silicone endo-tamponade in group 2 (*P* = 0.033). Visual recovery and macular configuration in eyes with PDR are not affected by whether the ILM is peeled before or after silicone oil tamponade.

## Introduction

Diabetic retinopathy is a common complication of diabetes mellitus and is a major cause of vision loss in middle-aged and elderly people^1^. Severe stage of diabetic retinopathy includes proliferative diabetic retinopathy (PDR), which is caused by the abnormal growth of new retinal blood vessels.

Vitrectomy is a surgical option for severe PDR. Silicone oil (SO) is frequently used for endo-tamponade at the end of vitrectomy for severe PDR with the expectation of retinal reattachment^2^. However, vision loss after uncomplicated surgery with SO tamponade has been reported^3^. There might be a relationship between migration of SO droplets into the retina and optic nerve and widespread loss of myelinated optic nerve fibers^4^. Thinning of the inner retina after SO tamponade has been identified by spectral-domain optical coherence tomography (SD-OCT)^5–7^. Ganglion cells can be damaged by direct contact with SO particles, which are located within the vitreous cavity and migrate into the retina^8^. Ganglion cells can also be indirectly damaged by phototoxicity due to SO transparency or by inflammatory damage caused by cytokines that are caught between the SO and retina^9^.

Peeling of the internal limiting membrane (ILM) has become a common maneuver in vitrectomy surgery. The ILM comprises the basement membranes of Müller cells and is located on the vitreous surface of the retina. Complete peeling of the ILM at the posterior vitreous has been reported to reduce the frequency of macular edema in eyes that have undergone vitrectomy for diabetic macular edema^10,11^. Furthermore, the prevention of macular pucker formation was reported in eyes with severe proliferative vitreoretinopathy^12–14^. However, ILM peeling may damage Müller cells and other cells in the retina^15,16^, causing changes in the configuration and structure of the macula, including retinal thinning^17–19^.

SO tamponade and ILM peeling may have a synergistic effect during vitrectomy. Kaneko et al. reported retinal thinning after combined treatment with SO tamponade and ILM peeling^20^. However, they included a small number of eyes, had a short follow-up duration, and did not report the effect on vision. This study aimed to determine the clinical outcomes of vitrectomy with SO tamponade for PDR at 1 year according to the timing of ILM peeling.

## Results

The medical records of 791 eyes (549 patients) who received PPV and SO tamponade treatment for PDR were retrospectively analyzed. A total of 63 eyes were included according to the eligible inclusion and exclusion criteria which is described in the method section. These were further divided into two groups: group 1 with ILM peeling during SO injection surgery (33 eyes, 30 patients) and group 2 with ILM peeling during SO removal surgery (30 eyes, 28 patients).

The patient characteristics are listed in Table 1. Patient’s age, sex, duration of diabetes, preoperative hemoglobin A_1C_, phakic-pseudophakic ratio, preoperative best-corrected visual acuity (BCVA), intraocular pressure (IOP), and spherical equivalent were similar between the groups. There was no significant between-group difference in the mean thickness of each retinal layer or in subfoveal choroidal thickness at baseline. The mean interval between injection and removal of SO was 4.78 ± 3.41 (range 1–9) months in group 1 and 4.82 ± 2.94 (range 1–9) months in group 2 (*P* = 0.637) without significant difference between-group.

**Table 1:** The baseline clinical characteristics and retinal layer measurements in different groups according to timing of ILM peeling.

	Group 1 (n = 33 eyes)	Group 2 (n = 30 eyes)	*P* value*
Age (years)	49.8 ± 11.0	50.0 ± 10.3	0.813
Sex (male to female ratio)	22 : 11	18 : 12	0.564
Duration of DM (years)	7.9 ± 5.9	7.2 ± 8.4	0.249
HbA_1C_ (%)	8.2 ± 1.9	8.6 ± 2.3	0.697
Phakic to pseudophakic ratio	17:0	15:1	0.311
Axial length (mm)	23.8 ± 2.0	23.16 ± 0.88	0.246
SE refractive error (D)	− 0.84 ± 1.91	− 0.16 ± 1.37	0.305
BCVA (Snellen)	0.22 ± 0.26	0.25 ± 0.30	0.567
IOP (mmHg)	16.59 ± 3.35	16.09 ± 3.17	0.488
Total retinal thickness (µm)	315.50 ± 69.45	311.04 ± 63.20	0.218
Outer retinal thickness (µm)	80.12 ± 5.99	82.91 ± 5.80	0.102
Inner retinal thickness (µm)	235.62 ± 67.56	223.25 ± 57.57	0.293
Gc-IPL thickness (µm)	54.12 ± 22.75	44.72 ± 16.01	0.101
RNFL thickness (µm)	22.31 ± 7.71	18.70 ± 9.18	0.117
Choroidal thickness (µm)	253.83 ± 72.79	261.26 ± 62.88	0.430
Duration of SO tamponade (months)	4.78 ± 3.41	4.82 ± 2.94	0.637

The data are presented as the mean ± standard deviation. Group 1, ILM peeling during SO injection surgery. Group 2, ILM peeling during SO removal surgery. The retinal layer thickness was measured in a 1-mm central macular area. *P < 0.05 was considered statistically significant, unpaired *t*-test. *BCVA* best-corrected visual acuity, *DM* diabetes mellitus, *Gc-IPL* ganglion cell-inner plexiform layer, *ILM* internal limiting membrane, *IOP* intraocular pressure, *RNFL* retinal nerve fiber layer, *SE* spherical equivalent, *SO* silicone oil.

### Between-group comparison of visual acuity and IOP

The preoperative and postoperative BCVA and IOP values are presented in Table 2. BCVA improved substantially after surgery in both the study groups with no significant between-group difference at 1 year after surgery (*P* = 0.151). The mean IOP was 16.59 ± 3.35 (range 10–24) mmHg in group 1 and 16.09 ± 3.17 (range 14–23) mmHg in group 2 at baseline; the values at 1 year after surgery were 17.00 ± 3.42 (range 13–23) mmHg (*P* = 0.551) and 17.05 ± 3.20 (range 12–23) mmHg (*P* = 0.263), respectively. No eye showed an increase in IOP of > 30 mmHg or instilled glaucoma eyedrops during the follow-up.

**Table 2:** Visual acuity and intraocular pressure measurements according to timing of ILM peeling.

	Group 1		Group 2		*P* value^‡^
		*P* value^†^		*P* value^†^	
**BCVA (Snellen)**					
Baseline	0.22 ± 0.26		0.25 ± 0.30		0.567
Before SOR surgery	0.21 ± 0.14	0.261	0.20 ± 0.17	0.269	0.186
3 months after SOR	0.44 ± 0.23	< 0.010*	0.43 ± 0.24	0.013*	0.549
6 months after SOR	0.47 ± 0.24	< 0.010*	0.47 ± 0.26	< 0.010*	0.194
1 year after SOR	0.54 ± 0.21	< 0.010*	0.48 ± 0.27	< 0.010*	0.151
**IOP (mmHg)**					
Baseline	16.59 ± 3.35		16.09 ± 3.17		0.488
Before SOR surgery	16.16 ± 2.63	0.318	16.72 ± 3.41	0.137	0.212
3 months after SOR	17.27 ± 3.53	0.770	16.31 ± 3.87	0.564	0.465
6 months after SOR	16.56 ± 3.35	0.902	16.52 ± 3.05	0.859	0.644
1 year after SOR	17.00 ± 3.42	0.551	17.05 ± 3.20	0.263	0.474

The data are shown as the mean ± standard deviation. Group 1, ILM peeling during SO injection surgery; Group 2, ILM peeling during SO removal surgery. ^†^At each time point vs. baseline, paired *t*-test. ^‡^Group 1 vs Group 2, independent *t*-test. *P < 0.05, statistically significant. *BCVA* best-corrected visual acuity, *ILM* internal limiting membrane, *IOP* intraocular pressure, *SOR* silicone oil removal.

### Between-group comparison of retinal thickness

The preoperative and postoperative retinal layer thicknesses are presented in Table 3. There was no considerable between-group difference in the thickness of the total, outer, or inner retinal layer or in that of the ganglion cell-inner plexiform layer (Gc-IPL) or retinal nerve fiber layer (RNFL) at any time point.

**Table 3:** Measurements of center macular retinal and choroidal thickness according to timing of ILM peeling.

	Group 1		Group 2		*P* value^‡^	Percent change^‡^
		*P* value^†^		*P* value^†^		
**Total retina (µm)**						
Baseline	315.50 ± 69.45		311.04 ± 63.20		0.218	
Before SOR	316.53 ± 75.69 (+ 1.97%)	0.932	331.08 ± 89.48 (+ 7.70%)	0.230	0.151	0.283
3 months after SOR	293.26 ± 59.27 (− 6.00%)	0.022*	288.32 ± 60.08 (− 3.56%)	0.083	0.518	0.951
6 months after SOR	283.69 ± 60.82 (− 11.90%)	0.001*	273.82 ± 57.73 (− 9.12%)	0.016*	0.421	0.550
12 months after SOR	280.08 ± 58.43 (− 11.88%)	0.001*	270.50 ± 56.28 (− 11.09%)	0.006*	0.241	0.866
**Outer retina (µm)**						
Baseline	80.12 ± 5.99		82.91 ± 5.80		0.102	
Before SOR	80.78 ± 7.76 (+ 1.01%)	0.594	80.68 ± 3.79 (− 2.77%)	0.207	0.902	0.090
3 months after SOR	83.56 ± 7.67 (− 2.56%)	0.135	82.48 ± 5.47 (− 0.54%)	0.507	0.426	0.690
6 months after SOR	82.11 ± 11.47 (+ 1.17%)	0.517	83.04 ± 4.52 (+ 0.14%)	0.968	0.653	0.703
12 months after SOR	83.16 ± 8.55 (+ 3.09%)	0.111	83.29 ± 5.55 (+ 0.40%)	0.829	0.160	0.322
**Inner retina (µm)**						
Baseline	235.62 ± 67.56		223.25 ± 57.57		0.293	
Before SOR	236.28 ± 73.51 (+ 3.22%)	0.743	250.72 ± 89.95 (+ 13.62%)	0.053	0.067	0.255
3 months after SOR	209.53 ± 56.55 (− 8.81%)	0.016*	204.96 ± 58.65 (− 7.17%)	0.670	0.149	0.801
6 months after SOR	201.69 ± 55.77 (− 16.08%)	0.009*	190.87 ± 56.86 (− 11.85%)	0.062	0.673	0.486
12 months after SOR	196.80 ± 56.89 (− 17.01%)	0.002*	187.58 ± 54.83 (− 14.26%)	0.022*	0.834	0.661
**Gc-IPL (µm)**						
Baseline	54.12 ± 22.75		44.72 ± 16.01		0.101	
Before SOR	52.90 ± 20.70 (+ 8.93%)	0.783	50.60 ± 19.56 (+ 17.29%)	0.430	0.213	0.577
3 months after SOR	45.50 ± 15.77 (− 5.56%)	0.031*	43.70 ± 19.05 (− 0.72%)	0.470	0.241	0.650
6 months after SOR	41.07 ± 18.05 (− 17.53%)	0.002*	42.26 ± 18.01 (− 4.33%)	0.203	0.200	0.208
12 months after SOR	41.66 ± 14.76 (− 16.64%)	0.004*	38.91 ± 15.67 (− 11.25%)	0.043*	0.767	0.583
**RNFL (µm)**						
Baseline	22.31 ± 7.71		18.70 ± 9.18		0.117	
Before SOR	23.68 ± 6.37 (+ 18.79%)	0.437	21.12 ± 8.45 (+ 26.79%)	0.231	0.248	0.611
3 months after SOR	19.06 ± 10.88 (− 1.16%)	0.309	16.88 ± 5.37 (− 0.49%)	0.216	0.418	0.967
6 months after SOR	17.15 ± 4.12 (− 16.06%)	0.001*	16.16 ± 4.49 (− 5.61%)	0.085	0.594	0.314
12 months after SOR	15.62 ± 3.86 (− 21.41%)	< 0.001*	15.04 ± 3.83 (− 11.81%)	0.028*	0.244	0.251
**Choroid (µm)**						
Baseline	253.83 ± 72.79		261.26 ± 62.88		0.432	
Before SOR	230.71 ± 67.33 (− 6.79%)	0.153	256.95 ± 80.84 (− 1.73%)	0.687	0.692	0.525
3 months after SOR	246.13 ± 57.78 (− 4.96%)	0.517	265.60 ± 68.28 (+ 2.88%)	0.964	0.283	0.403
6 months after SOR	239.53 ± 57.86 (− 2.47%)	0.233	259.60 ± 71.13 (+ 0.72%)	0.689	0.419	0.733
1 year after SOR	235.52 ± 49.79 (− 2.89%)	0.148	251.62 ± 74.21 (+ 0.77%)	0.593	0.364	0.726

The values are presented as the mean ± standard deviation (percent change). Group 1, ILM peeling during SO injection surgery. Group 2, ILM peeling during SO removal surgery. ^†^At each time point vs. baseline, paired *t*-test. ^‡^Group1 vs Group2, independent *t*-test. *P < 0.05, statistically significant. *Gc-IPL* ganglion cell-inner plexiform layer, *ILM* internal limiting membrane, *RNFL* retinal nerve fiber layer, *SOR* silicone oil removal.

In group 1, the central macular thickness (CMT) increased slightly but not significantly (*P* = 0.932) during the interval (the SO endo-tamponade period) between the first vitrectomy (with SO tamponade and ILM peeling) and second vitrectomy. Furthermore, there was no significant change in the outer retinal, inner retinal, Gc-IPL, or RNFL thickness (*P* = 0.594, 0.743, 0.783, and 0.437, respectively). Twelve months after silicone removal surgery, there were significant decreases from baseline in the thicknesses of the total and inner retina layers and the Gc-IPL and RNFL (all *P* < 0.050) but not in the outer retinal layer thickness (*P* = 0.111).

In group 2, the CMT increased slightly during the SO endo-tamponade period, although the change was not considerable. The changes in outer retinal, inner retinal, Gc-IPL, and RNFL thicknesses were also not substantial; however, 12 months after SO removal surgery, the total retina, inner retina, Gc-IPL, and RNFL thicknesses decreased substantially. Nevertheless, the thickness of the outer retinal layer at 12 months after SO removal was similar to that at baseline (*P* = 0.829).

### Between-group comparison of subfoveal choroidal thickness

There was no considerable between-group difference in choroidal thickness at any time point. The choroidal thickness decreased, but not significantly, during SO endo-tamponade in study group 1 (*P* = 0.153) and 2 (*P* = 0.687), respectively. Moreover, no significant change was noted at 12 months after SO removal surgery (Table 3).

### Between-group comparison of incidence of epiretinal membrane (ERM)

ERM was not observed on OCT in either of the study group before surgery; however, it was detected after SO injection surgery in both the groups, with an incidence of 3% (n = 1) in group 1 and 20% (n = 6) in group 2 (*P* = 0.033). There was a borderline significant increase in the thickness of the inner retinal layer from 223.25 ± 57.57 µm to 250.72 ± 89.95 µm in group 2 (*P* = 0.053). ERM was located in the extrafoveal area (outside the area of ILM peeling) in group 1 eyes and was peeled during the secondary vitrectomy for SO removal (Table 4).

**Table 4:** Central macular thickness and macular ERM according to timing of ILM peeling.

	Group 1	Group 2	*P* value
**Baseline**			
CMT (µm)	315.50 ± 69.45	311.04 ± 63.20	0.218^†^
Macular ERM	0% (0/33)	0% (0/30)	
**SO endo-tamponade**			
Duration of tamponade (months)	4.78 ± 3.41	4.82 ± 2.94	0.637^†^
CMT (µm)	316.53 ± 75.69	331.08 ± 89.48	0.151^†^
Macular ERM	3% (1/33)	20.0% (6/30)	0.033^‡,^*
**Final**			
CMT (µm)	280.08 ± 58.43	270.50 ± 56.28	0.241^†^
Macular ERM	0% (0/33)	0% (0/30)	

The data are shown as the mean ± standard deviation or as the percentage (number). Group 1, ILM peeling during SO injection surgery. Group 2, ILM peeling during SO removal surgery. ^†^unpaired *t*-test. ^‡^chi-squared test. *P < 0.05, statistically significant. *CMT* central macular thickness, *ERM* epiretinal membrane, *ILM* internal limiting membrane, *SO* silicone oil.

No cases of ERM were observed in either of the study group at the 1-year visit. After the second vitrectomy for SO removal, there was a significant decrease in the CMT in both groups 1 and 2 (*P* = 0.001 and *P* = 0.006, respectively) as compared with baseline.

### Correlation between the final postoperative BCVA and the other multiple variables

Multiple regression analysis was performed to analyze the correlation between the final postoperative visual acuity and other multiple variables including the change in retinal layer thickness, SO tamponade period, ILM peeling timing (binary variable), age and gender in both group 1 and 2. As a result of the analysis, the final postoperative BCVA showed a significant negative correlation with the amount of thinning in the inner retinal layer (*r* = − 0.418, *P* = 0.008) and total retinal layer (*r* = − 0.371, *P* = 0.019). However, there was no significant correlation between the SO tamponade period (*r* = 0.281, *P* = 0.079), changes in choroidal layer thickness (*r* = − 0.290, *P* = 0.082), changes in outer retinal layer thickness (*r* = − 0.095, *P* = 0.565), gender (*r* = − 0.055, *P* = 0.717), age (*r* = − 0.018, *P* = 0.904) and the final BCVA.

## Discussion

In this study, we investigated the combined effect of silicone tamponade and ILM peeling in vitrectomy surgery on retinal thickness and visual function. Moreover, the effect of timing of ILM peeling (before SO tamponade or after SO removal) on clinical outcomes and retinal and choroidal thickness in eyes with PDR at 1 year after surgery were also observed. We found that the thickness of the inner retinal layer decreased in the eyes that underwent ILM peeling with SO tamponade surgery, with no considerable change in the outer retinal layer or subfoveal choroidal thickness. The timing of ILM peeling did not affect the final visual outcome or degree of macular thinning at 1 year after SO removal surgery. This is the first report on the long-term changes in visual acuity and thickness of the retinal and choroidal layers after ILM peeling and SO tamponade/removal surgery in eyes with PDR.

The thickness of the inner retinal layer, including the Gc-IPL and RNFL, decreased after combined SO tamponade and ILM peeling in both our study groups. Retinal thinning after silicone tamponade or ILM peeling has been reported previously^5–7,17–19^. Kaneko et al. reported that macular retinal thinning was greater in eyes treated with ILM peeling and SO tamponade^20^. However, their report has some limitations, which include a small sample size of eight eyes that underwent SO(+)/ILM peeling(+) and were on a short follow-up of only 3–7 months after the first vitrectomy surgery. Moreover, there was no detailed analysis of whether the timing of ILM peeling affected the clinical outcomes of vitrectomy with SO tamponade. In our study, the retinal layer thickness (total, inner, Gc-IPL, RNFL) decreased from baseline in both the study groups, with no considerable between-group difference.

There are several mechanisms by which thinning of the inner retinal layer could occur after SO tamponade or ILM peeling assisted by indocyanine green (ICG) dye staining during vitrectomy surgery. SO tamponade, ILM peeling, and ICG dye staining have all been reported to cause retinal thinning^5–7,17–19,21^. First, SO tamponade may have an indirect toxic effect on the retina by causing local changes in ion and cytokine concentrations. For example, the potassium concentration may increase because of failure of potassium siphoning by Müller cells. Furthermore, expression of proinflammatory cytokines is increased in the fluid between the SO and retinal surface^22,23^. Second, ICG dye may damage retinal neurosensory and ganglion cells, resulting in retinal thinning^21^. Third, ILM peeling may result in mechanical impairment of Muller glial cells^10,23,24^. Moreover, without the ILM, the retina might be directly exposed to the fluid between the retina and SO, which has a high cytokine content, resulting in even more severe damage^5,10,25,26^. Kaneko et al. postulated that the ILM has a protective role during SO tamponade^25^. However, there have been some histological studies showing that the pores and thin points in the ILM may allow movement of cells and cytokines from the retina to the vitreous^24,26^. Furthermore, SO contains low-molecular-weight components that might penetrate into the retina independent of the ILM. However, in our study, there was no significant between-group difference in the final visual acuity or retinal layer thickness despite the retina being directly exposed to SO in group 1 alone and for an average of 3–4 months during the SO endo-tamponade period. More studies are required to determine if SO tamponade and ILM peeling have a synergistic effect on retinal thinning.

Subfoveal choroidal thickness decreased after SO injection during vitrectomy surgery in both our study groups, and was maintained at the 1-year postoperative visit. Although this change was not statistically significant, there has been a report of significant choroidal thinning after injection of SO^27^, which could be related to changes in ion and fluid flow between the retina, retinal pigment epithelium, and choroid. Our study included a relatively small number of cases, and we did not take EDI-OCT measurements. Therefore, further studies are required to analyze the choroidal thickness after SO injection.

We observed an improvement in final BCVA after surgery in both the groups, with no substantial between-group difference in the amount of improvement. There was a correlation between thinning of the inner retinal layer and all retinal layers with the final BCVA at 1 year after surgery. A significant correlation between vision loss and inner retinal thinning in SO-filled eyes has been reported previously^5^. Our sub-analysis also revealed a significant correlation of inner retinal layer thickness with the Gc-IPL and RNFL. Thinning of the Gc-IPL, including the ganglion cell bodies and dendrites, and of the RNFL containing the ganglion cell axons, would affect vision. However, further studies with a larger sample size are needed to validate our results.

In this study, the incidence of macular ERM during the SO endo-tamponade period was significantly greater in group 2 than in group 1 (20% [6/30 eyes] vs. 3% [1/33 eyes]). Furthermore, the inner retinal layer thickness increased during this period in group 2, although the difference was not statistically significant (*P* = 0.053). Removal of the ILM appears beneficial in terms of allowing complete peeling of the posterior surface of the vitreous and the scaffold of proliferating astrocytes^10,28^, which prevents macular pucker^12,13,25^. Fewer cases of ERM and macular edema have been observed after vitrectomy with ILM peeling in eyes with PDR^13,14^. We found that ILM peeling in eyes with PDR prevented ERM during the SO endo-tamponade period, which is in accordance with the previous studies. After SO removal and ILM peeling, ERM was not observed during the 1 year of follow-up in either of our study groups.

Our study has some limitations. First, the study was retrospective and lacked randomization with selection bias. Second, although the automated segmentation of the Spectralis OCT showed excellent repeatability and reproducibility, there was a shift in the ETDRS [Early Treatment Diabetic Retinopathy Study] foveal center in some OCT images after surgery. Therefore, we checked every image and shifted the foveal center manually in some cases. Third, the sample size was relatively small. However, to the best of our knowledge, there have been no similar studies that have included a larger sample size or a longer follow-up period.

In conclusion, this study found that the thickness of the inner retinal layer, including the Gc-IPL and RNFL, decreased in eyes that underwent combined SO tamponade and ILM peeling for PDR. The timing of ILM peeling did not significantly affect the final visual outcome or degree of macular thinning at 1 year after SO removal.

## Methods

This retrospective study was approved by the Institutional Review Board of Hangil Eye Hospital and adhered to the tenets of the Declaration of Helsinki. The requirement to obtain informed consent from study participants was waived by the institutional review board given the retrospective nature of the study.

### Subjects

All patients in the study underwent two-stage vitrectomy (injection of SO followed by its removal) with ILM peeling performed by the same surgeon (DDH) for severe PDR at Hangil Eye Hospital from January 2014 to January 2019. Phacoemulsification and posterior chamber IOL insertion was performed along the 1st vitrectomy in all eyes except one preoperative pseudophakic eye in group 2. The inclusion criteria were (1) severe PDR with tractional retinal detachment and persistent bleeding indicated for surgery, (2) no unexpected intraoperative or postoperative complications, (3) SO evacuated no more than 9 months after primary SO tamponade surgery, and (4) SD-OCT performed before and after surgery. The following exclusion criteria were applied: preoperative foveal traction or detachment because of inaccurate measurement of retinal thickness using OCT; preoperative thick vitreous hemorrhage which affect the OCT image quality (signal intensity < 25); SO endo-tamponade for longer than 9 months; any ocular or systemic disorder that could affect retinal thickness (e.g., glaucoma, optic nerve disease, epiretinal membrane, or age-related macular degeneration); additional surgery required to treat complications, such as retinal detachment or proliferation of membranes; history of ocular surgery including vitrectomy.

### Surgical methods

Three-port 25-gauge transconjunctival sutureless vitrectomy was performed using the Constellation Vision Surgical System (Alcon Surgical, Fort Worth, TX, USA). Core vitrectomy and peripheral vitreous shaving with removal of vitreous traction and proliferative membranes were performed first, and endolaser photocoagulation was applied to the retina. SO (5500 Centistoke, Arcadophta, Toulouse, France) was injected after fluid-air exchange. SO was used at the surgeon’s discretion in cases with vigorous bleeding or severe tractional retinal detachment caused by fibrovascular membranes or their removal.

The eyes were divided according to whether ILM peeling was performed during SO injection surgery (group 1) or during SO removal surgery (group 2). All ILM peeling was assisted with triamcinolone (MaQaid^®^, Wakamoto Pharmaceutical Co., Ltd., Tokyo, Japan). In all cases, an area of ILM measuring approximately 6 mm in diameter (equivalent to the entire ETDRS sector area) was peeled at the posterior pole of the retina. The surgery was completed after confirmed absence of any traces of ILM or macular hole using intraoperative OCT (RESCAN 700, Zeiss, Oberkochen, Germany) in all patients.

### Ophthalmic examinations

Before surgery, all subjects underwent a complete ophthalmic examination, including measurement of BCVA, IOP, slit-lamp examination, dilated fundus examination, automated keratometry, specular microscopy, and SD-OCT (Spectralis, Heidelberg Engineering, Heidelberg, Germany).

BCVA, IOP, and OCT measurements and slit-lamp and dilated fundus examination findings were recorded after injection of SO and at 3, 6, and 12 months after its removal. The main outcome measures were changes in thickness of the retinal layers and BCVA, IOP, and OCT findings at follow-up visits.

### Measurement of retinal and choroidal thicknesses

Macular thickness was measured using the Spectralis OCT, which automatically detects the center position of the fovea based on the ETDRS chart. The inbuilt Spectralis software, Heidelberg Eye Explorer (version 6.0.9.0) automatically segments and measures the thickness of each retinal layer.

Total, outer, inner retinal, Gc-IPL, and RNFL thicknesses were measured within 1 mm of the central macular subfield by OCT. The thickness of the outer retinal layer was measured from Bruch’s membrane to the external limiting membrane and the inner retinal layer was measured from the external limiting membrane to the RNFL. Choroidal thickness was measured in the subfoveal area using a built-in caliper. The degree of change in each layer was then analyzed. Kaneko et al. highlighted the importance of the relative reduction in retinal thickness between preoperative and postoperative measurements^20^. Therefore, we investigated both the actual size and percentage change in retinal thickness. The percentage change was obtained by dividing the difference between the preoperative and postoperative thicknesses by the preoperative thickness.

Preoperative OCT measurements were obtained 1 week before the primary vitrectomy surgery and postoperative measurements at 1 week before and 3, 6, and 12 months after the second vitrectomy surgery (for removal of SO). Patients with poor-quality OCT images (signal strength ≤ 7) were excluded from the study.

### Statistical analyses

The data for retinal layer and choroidal thickness were confirmed to be normally distributed using the Shapiro–Wilk test. Baseline clinical characteristics were compared between groups 1 and 2 using the independent *t*-test and Pearson’s chi-squared test. The correlation between the final BCVA and the multiple variables were examined using multiple regression analysis. The frequency of ERM was compared between the groups using the chi-squared test. All statistical analyses were performed using SPSS software (version 25.0; IBM Corp., Armonk, NY, USA). A *P*-value < 0.050 was considered statistically significant.

## Data Availability

The data are not available for public access because of patient privacy concerns, but are available from the corresponding author upon reasonable request.
